# On Flat Foot and Its Treatment

**Published:** 1897-01-23

**Authors:** J. Lacy Firth

**Affiliations:** Assistant Surgeon, Bristol General Hospital.


### ON FLAT FOOT AND ITS TREATMENT.

By J. Lacy Firth, M.D., M.S.(Lond.), F.R.C.S., Assistant Surgeon, Bristol General Hospital.

Acquired flat foot or talipes valgus is a deformity of frequent occurrence, and may give rise to very distressing symptoms. Congenital, as distinguished from acquired forms of talipes valgus exist, but are uncommon. Of the acquired deformity there are varieties, determined by their cause. The static variety (1) is the one we shall chiefly consider, but the others may be briefly noticed. (2) Traumatic flat foot is produced by violence, either immediately or remotely. A fall from a height upon the feet may rupture the ligaments which maintain the arch of the foot, and flatten it immediately, or may so damage the ligaments that subsequently they remain too weak, and yield under the body weight. The violence may in the first instance fracture the fibula or some other bone near the ankle, and a deformity may be left permanently, which throws more strain than normal upon the plantar arch in standing or walking, and so causes flattening. Thus traumatic flat foot is a common cause of pain and weakness after badly united Pott's fractures. (3) Paralytic flat foot may result from infantile palsy. In these cases the tibial muscles (anticus and pisticus) and the flexor longus hallucis have lost the power they normally possess of bracing up the inner arch of the foot. (4) Spastic flat foot is the result of the spasm of muscles which certain nervous diseases cause. The spasm pulls the foot into the bad position and overcomes the muscular and ligamentus supports of the arch. (5) Rickety flat foot is caused by the muscular weakness that disease so often produces, and is favoured by the various deformities of the legs which may arise, knock-knee, bowed legs, and curved tibiæ. (6) Hysterical flat foot is sometimes met with. (7) Flat foot may result from diseases of the ankle joint, *e.g.*, rheumatism.

Static flat foot is most frequently met with in people between the ages of twelve and twenty, and is caused by the yielding, under the strain of the body weight, of those muscles and ligaments which support the inner longitudinal or instep arch of the foot. Any cause which tends to throw more strain upon the supports of the arch, if it be not met by proportionate increase of their strength, will produce flattening of the sole. Such causes are prolonged standing, especially with the feet abducted, undue weight carrying, inequality in the length of the legs, and deformities, which cause the transmission of the body-weight to the feet in a more inward direction than usual, such as knock-knee, genu varum, and curved tibiæ. Again, all conditions which weaken primarily the supports of the arch have the same effect. Weakness of the muscular supports may be due to general debility in convalescence from an acute illness, or from rapid growth, or from other constitutional states, *e.g.*, anæmia. Badly-shaped boots come in this class of causes, for they interfere with the proper exercise of the muscles, and, if they produce hallux valgus, they specially weaken the flexor longus hallucis by altering the direction and line of action of its tendon.

Two muscles are especially concerned with the support of the arch of the instep, the tibialis posticus and the flexor longus hallucis; and two ligaments, the inferior calcaneo-scaphoid and the interosseous calcaneo-astragaloid. When the feet are abducted, a movement which takes place in subastragaloid and the median tarsal joints chiefly, the muscles mentioned are relaxed and may be strained, and the ligaments are relaxed. People who stand long like to abduct the feet for the purpose of resting the fatigued muscles. Thus in long standing the feet are frequently thrown into that position in which the least support is given to the arch, and most strain is borne by the ligaments. Further, in standing with the feet at right angles to the body (*i.e.*, adducted) the body-weight is transmitted downwards and slightly inwards through the astragalus almost entirely to the heels (os calcis). But when the feet are abducted the astragalus are moved slightly forwards, and with them the direction of transmission of the body-weight is moved forwards, and more pressure is brought to bear upon the inner arch of the foot. The head of the astragalus sinks a little, and the inner border of the foot is lengthened. The component of the body-weight directed forwards is transmitted, in part, to the anterior and inner portion of the upper surface of the os calcis, and, being applied there, tends to rotate the os calcis in its long antero-posterior axis inwards. Thus the inner surface of the os calcis and the sustentacular tali have a tendency to assume a downward rather than the normal inward direction, and the outer tuberosity of the bone tends to assume an upward and outward rather than the normal downward direction. In abduction the forces that tend this way are all increased, and, if the ligaments yield, the os calcis actually assumes the rotated position. The yielding also allows the head of the astragalus to sink more and more downwards, and so in severe cases of flat foot it may produce a convexity downwards where the concavity of the instep should exist. Thus, in standing and in walking, the adducted position of the foot is the strong position, for in it the body-weight is transmitted mostly to the heel, and the muscles which assist in maintaining the arch are active and can more effectually resist the small downwardly and inwardly acting component of the body-weight. Thus it is that the most successful treatment for flat foot is that which strengthens the muscles supporting the arch, and places the foot in such a position that the least strain has to be borne by that arch. Knock-knee and curved tibiæ tend to produce flat foot, because with these deformities the body-weight is thrown more to the inner side of the foot. In some forms of curved tibiæ and in genu varum the malleolar arch is tilted upwards on the inner side and looks downwards and inwards instead of directly downwards, the astragalus embraced by the arch at the ankle-joint has a similar direction, and in consequence tends to slip upon the os calcis inwards and to displace that bone outwards. In hallux valgus the tendon of the flexor longus hallucis is carried outwards, and has less power to maintain the arch of the inner border of the foot as a consequence.

